# MAPPING 15-YEAR DEPRESSIVE SYMPTOM TRANSITIONS IN LATE LIFE: A POPULATION-BASED COHORT STUDY

**DOI:** 10.1093/geroni/igae098.1157

**Published:** 2024-12-31

**Authors:** Federico Triolo, Davide Liborio Vetrano, Caterina Trevisan, Linnea Sjöberg, Amaia Calderón-Larrañaga, Martino Belvederi Murri, Laura Fratiglioni, Serhiy Dekhtyar

**Affiliations:** Aging Research Center, Karolinska Institutet, Stockholm, Stockholms Lan, Sweden; Karolinska Institutet, Stockholm, Stockholms Lan, Sweden; Karolinska Institutet, Stockholm, Stockholms Lan, Sweden; Aging Research Center, Karolinska Institutet, Stockholm, Stockholms Lan, Sweden; Karolinska Institutet, Stockholm, Stockholms Lan, Sweden; Institute of Psychiatry, Ferrara, Emilia-Romagna, Italy; Aging Research Center, Karolinska Institutet, Stockholm, Stockholms Lan, Sweden; Aging Research Center, Karolinska Institutet, Stockholm, Stockholms Lan, Sweden

## Abstract

As the longitudinal course of late-life depression remains unclear, we aimed to describe transitions along the depression continuum, and identify factors associated with specific transition patterns. We analysed 15-year longitudinal data on 2745 dementia-free persons aged 60+ from the Swedish National Study on Aging and Care in Kungsholmen. Depression (minor and major) was diagnosed according to DSM-IV-TR; subsyndromal depression (SSD) was operationalized as the presence of ≥2 symptoms without depression. Multistate survival models were used to map depression transitions, including death, and to examine the association of psychosocial (social network, connection, and support), lifestyle (smoking, alcohol consumption, and physical activity), and clinical (somatic disease count) factors with transition patterns. Over the follow-up, 19.1% had ≥1 transitions across depressive states, while 6.5% had ≥2. Each additional somatic disease was associated with a higher hazard of progression from no depression to SSD (HR 1.09; 1.07-1.10) and depression (HR 1.06; 1.04-1.08), but also with a lower recovery (HRSSD-NoDep 0.95; 0.93-0.97; HRDep-NoDep 0.96; 0.93-0.99). Physical activity was associated with an increased hazard of recovery to no depression from SSD (HR 1.49; 1.28-1.73) and depression (HR 1.20; 1.00-1.44), while richer social network was associated with both higher recovery (HRSSD-NoDep 1.44; 1.26-1.66; HRDep-NoDep 1.51; 1.34-1.71), and lower progression hazards (HRNoDep-SSD 0.81; 0.70-0.94; HRNoDep-Dep 0.58; 0.46-0.73; HRSSD-Dep 0.66; 0.44-0.98). Older people may present with heterogeneous depressive trajectories. Targeting the accumulation of somatic diseases and enhancing social interactions may be appropriate for both depression prevention and burden reduction, while promoting physical activity may benefit recovery from depressive disorders.

